# Functional Analysis of *Cotton Leaf Curl Kokhran Virus*/Cotton Leaf Curl Multan Betasatellite RNA Silencing Suppressors

**DOI:** 10.3390/biology4040697

**Published:** 2015-10-23

**Authors:** Muhammad Saeed, Rob W. Briddon, Athanasios Dalakouras, Gabi Krczal, Michael Wassenegger

**Affiliations:** 1 RLP AgroScience GmbH, AlPlanta-Institute for Plant Research, Breitenweg 71, Neustadt D-67435, Germany; E-Mails: athanasios.dalakouras@agroscience.rlp.de (A.D.); gabi.krczal@agroscience.rlp.de (G.K.); michael.wassenegger@agroscience.rlp.de (M.W.); 2 National Institute for Biotechnology and Genetic Engineering, Jhang Road, PO Box 577, Faisalabad 38000, Pakistan; E-Mail: rob.briddon@gmail.com; 3 Centre for Organismal Studies (COS) Heidelberg, University of Heidelberg, Im Neuenheimer Feld 360, Heidelberg D-69120, Germany

**Keywords:** begomovirus, betasatellite, post-transcriptional gene silencing, siRNA, RNA silencing suppressor

## Abstract

In South Asia, Cotton leaf curl disease (CLCuD) is caused by a complex of phylogenetically-related begomovirus species and a specific betasatellite, Cotton leaf curl Multan betasatellite (CLCuMuB). The post-transcriptional gene silencing (PTGS) suppression activities of the transcriptional activator protein (TrAP), C4, V2 and βC1 proteins encoded by *Cotton leaf curl Kokhran virus* (CLCuKoV)/CLCuMuB were assessed in *Nicotiana benthamiana*. A variable degree of local silencing suppression was observed for each viral protein tested, with V2 protein exhibiting the strongest suppression activity and only the C4 protein preventing the spread of systemic silencing. The CLCuKoV-encoded TrAP, C4, V2 and CLCuMuB-encoded βC1 proteins were expressed in *Escherichia coli* and purified. TrAP was shown to bind various small and long nucleic acids including single-stranded (ss) and double-stranded (ds) RNA and DNA molecules. C4, V2, and βC1 bound ssDNA and dsDNA with varying affinities. Transgenic expression of C4 under the constitutive 35S *Cauliflower mosaic virus* promoter and βC1 under a dexamethasone inducible promoter induced severe developmental abnormalities in *N. benthamiana*. The results indicate that homologous proteins from even quite closely related begomoviruses may differ in their suppressor activity and mechanism of action. The significance of these findings is discussed.

## 1. Introduction

In plants, RNA silencing is induced by the presence of double-stranded RNA (dsRNA). Sources of dsRNA are, among others, replication intermediates of viruses and viroids, transcription of inverted repeats, stress-induced overlapping antisense transcripts and RNA-DIRECTED RNA POLYMERASE (RDR) transcription of aberrant transcripts [1]. The DICER-LIKE (DCL) endonucleases, DCL2, DCL3, and DCL4 process dsRNA into 22-, 24-, and 21-nucleotides (nt) long small interfering RNAs (siRNAs), respectively [2]. Mature siRNAs exhibit 2 nt overhangs at the 3' end and are stabilized through 3' end methylation by HUE ENHANCER1 (HEN1) [3]. Depending on their 5' terminal nucleotide, siRNAs are loaded onto specific ARGONAUTE (AGO) proteins. Only one siRNA strand, the guide strand, is incorporated [4]. In general, 21 nt siRNAs are loaded onto AGO1 and mediate cleavage of complementary RNA in a process termed post-transcriptional gene silencing (PTGS) [5]. In the nucleus, 24-nt siRNAs are mainly incorporated into AGO4 and are involved in RNA-directed DNA methylation (RdDM) of cognate sequences [6]. Transitive silencing occurs when an initial pool of (primary) siRNAs, directed against the initial target sequence of a cleaved RNA, induces the production of secondary siRNAs that map to regions flanking the primary target site upstream (5') and downstream (3') [7]. The indispensable role of RDR6 in this process, copying the target transcript into dsRNAs, is well established [1,8,9,10,11]. Importantly, PTGS is not cell autonomous, it can spread to neighbouring cells and to distant parts of the plant. In cell-to-cell silencing, 21 nt siRNA duplexes appear to move through plasmodesmata and initiate PTGS at a distance of about 10–15 neighbouring cells [12,13]. Systemic silencing is mediated via the phloem-mediated transfer of RNA molecules (of an as yet unknown nature) to distant parts of the plant in a typical source-to-sink manner [14,15,16]. RNA silencing in plants has, at least in part, evolved as an antiviral defence mechanism, and plants deficient in RNA silencing genes are more susceptible and exhibit more severe symptoms [17]. As a counter-defence strategy viruses encode proteins (known as RNA silencing suppressors (RSS)) that suppress host RNA silencing and facilitate viral proliferation [18].

Viruses of the family *Geminiviridae* have circular single-stranded (ss) DNA genomes that are encapsidated in characteristic twinned icosahedral (geminate) capsids [19]. The most abundant geminiviruses are those that are transmitted by the whitefly *Bemisia tabaci* and are classified in the genus *Begomovirus*. A majority of the begomoviruses originating from the New World (NW) have genomes consisting of two components, known as DNA A and DNA B, both of which are required for virus infectivity. Recently, a single monopartite begomovirus (with a genome consisting of only a homolog of DNA A components of bipartite viruses) has been identified in South America [20]. In the Old World (OW), however, although a small number of bipartite viruses have been identified, the majority of begomoviruses have monopartite genomes. The genomes of monopartite, and DNA A components of bipartite, begomoviruses encode in the virion-sense the coat protein (CP) which is required for encapsidation, transmission by insects, and movement in plants and the V2 protein, homologous to the AV2 protein for bipartite begomoviruses, is involved in virus movement and may act as an RSS. The complementary-sense strand encodes (i) the replication-associated protein (Rep); a rolling-circle replication initiator protein; (ii) the transcriptional-activator protein (TrAP) which is involved in the up-regulation of late viral gene expression, modulates host gene expression and suppresses gene silencing; (iii) the replication enhancer protein (REn) which is involved in creating a cellular environment that is conducive to virus replication and (iv) the C4 protein which may be a pathogenicity determinant and an RSS [21,22].

Although a small proportion of monopartite begomoviruses may be truly monopartite, the majority of monopartite OW begomoviruses associate with a class of ssDNA satellites known as betasatellites (earlier known as DNA β; [23,24]). These satellites are small (~1.4 kb), circular ssDNA molecules that have only been identified in the OW and which are dependent upon a helper virus for replication, movement within and transmission between plants [23]. Betasatellites encode a single gene that typically encodes a protein of 118 amino acids referred to as βC1. The βC1 protein is a pathogenicity determinant, an RSS, binds DNA and possibly mediates virus movement in plants [25,26,27].

Cotton leaf curl disease (CLCuD) is the most significant biotic constraint to cotton production across most of Pakistan and northwestern India [28]. The disease first appeared in an epidemic form in Pakistan in 1991–1992. At that time it was shown to be caused by several monopartite begomoviruses (nine distinct species were ultimately identified, including *Cotton leaf curl Kokhran virus* [CLCuKoV] and *Cotton leaf curl Multan virus* (CLCuMuV); reviewed by Sattar *et al.* (2013) [28]) which were often found in multiple infections [29], and together with Cotton leaf curl Multan betasatellite (CLCuMuB; [30]). The TrAP, C4 and V2 proteins of CLCuMuV and the βC1 protein of CLCuMuB have previously been shown to have RSS activity [26]. In the present study, the same three proteins encoded by CLCuKoV were examined for their RSS activity. The CLCuMuB βC1 was additionally examined in more detail than in previous studies. The results show even quite similar viruses to have RSS proteins with distinct activities. The significance of the results are discussed.

## 2. Materials and Methods

### 2.1. Generation of Constructs

The genes encoding the TrAP, C4, V2 of CLCuKoV (accession no. AJ496286) and βC1 of CLCuMuB (AJ298903) were PCR-amplified with specific primers (Supplementary Table S1). The PCR products for the TrAP, C4, and V2 genes were restricted with *Eco*RI and *Sal*I, whereas the βC1 PCR product was restricted with *Eco*RI and *Hin*dIII. The cleaved PCR products were cloned into the identically cleaved binary vector pPCV702SM [6] to yield the constructs pPCV702SM-TrAP, pPCV702SM-C4, pPCV702SM-V2, and pPCV702SM-βC1. The plasmid pBAR-P19, containing the p19 sequence of *Tomato bushy stunt virus* (TBSV) genomic RNA, “statice” isolate (AJ249740, [31]) was used as template in two PCR reactions. In the first PCR, the primers 5'-GAATTCGAGCTCGGTACCAT-3' and 5'-AAGCTTGTTCCCTAGCATCG-3' were used, and the 61 bp amplicon was *EcoR*I/*Hind*III cloned into pPCV702SM [6], generating pPCV702SM-P19-5'. In the second PCR, primers: 5'-AAGCTTATGGTGAACGTTGG-3' and 5'-AAGCTTACTCGCTTTCTTCTTTG-3' were used, and the 486 bp amplicon was *Hind*III cloned into pPCV702SM-P19-5', generating pPCV702SM-p19. Additionally, the CLCuMuB βC1 gene was cloned as an *Xho*I/*Spe*I fragment into the expression vector pTA7001 [32], containing a dexamethasone inducible promoter, to produce pTA700-βC1. The integrity of all constructs was confirmed by sequencing. The production of pPCV702SM constructs for the expression of the full-length green fluorescence protein (GFP) gene (pPCV702SM-GFP5; [33], the middle 139 nt of the GFP gene (coordinates 231–369; pPCV702SM-GpG; [34]) and the β-glucuronidase (GUS) gene (pPCV702SM-GUS; [34]) have been described previously.

### 2.2. Plants and Plant Transformation

Transgenic *N. benthamiana*, line 16c, carrying a GFP transgene has been described previously [35]. The pPCV702SM or pTA700 constructs were individually introduced into *Agrobacterium tumefaciens* GV3101 by electroporation. *N. benthamiana* was transformed with these constructs as previously described [6]. Transgenic shoots were selected on (½ MS salts, 3% sucrose) supplemented with 100 μg/mL of kanamycin (pPCV702SM constructs) or 30 μg/mL of hygromycin (pTA700-βC1) and grown at 25 to 27 °C under artificial light (150 μEs^−1^·m^−2^) with a 16 h photoperiod. Transgenic plants were transferred to potting soil and were maintained in the glasshouse at 25 °C to 30 °C.

### 2.3. Agro-Infiltration Patch Assay

*A. tumefaciens* cultures harbouring pPCV702SM constructs were grown for 36 to 48 h. For sense PTGS (S-PTGS), pPCV702SM-GFP5 (expressing a functional GFP protein) and putative RSSs were each adjusted to an OD_600_ of 1.0 and mixed in equal proportions. For inverted repeat PTGS (IR-PTGS), pPCV702SM-GpG, pPCV702SM-GFP5 and putative RSSs were adjusted to OD_600_ of 1.0 and mixed in equal proportions. The *Agrobacterium* cultures were pelleted by gentle centrifugation, mixed in appropriate amounts of infiltration buffer [10 mM 2-(*N*-morpholino)ethanesulfonic acid (MES), pH 5.5, 10 mM MgCl_2_, 100 µM acetosyringone] and incubated at room temperature for between 2 h and 16 h. Fully expanded leaves of the 16c line were infiltrated with the *Agrobacterium* cultures. Infiltrated leaves were photographed at four days post infiltration under UV light in the dark and the leaves were harvested. Total RNA was isolated from leaves using peqGOLD TriFast^TM^ (Peqlab, Erlangen, Germany) reagent according to manufacturer’s recommendations. The presence of GFP mRNA was detected by northern blot hybridization, using the full-length ^32^P-labeled GFP probe as previously described [34]. Total RNA (10 µg for GFP mRNA detection, 25 µg for siRNA detection in S-PTGS, 20 µg for primary siRNA and 40 µg for secondary siRNA detection in IR-PTGS) were separated on 15% polyacrylamide (for siRNA) and on 1% agarose (for mRNA) and transferred onto positively charged nylon membranes (Roche Diagnostics GmbH, Mannheim, Germany). SiRNA produced in S-PTGS as well as mRNA were detected using the full-length ^32^P-labeled GFP probe. Primary siRNA were detected using the middle 139 bp long fragment of the GFP cDNA (U87973, coordinates 251–389, F probe) and secondary siRNA were detected using the 227 bp long N-terminal (U87973, coordinates 24–250, G probe) and the 269 bp long C-terminal fragments (U87973, coordinates 390–658, P probe). The G and P probe constituted the G/P probe. The sequence of primers used to amplify probes is given in Supplemental Table S1.

### 2.4. Expression and Purification of Recombinant Proteins

The full-length TrAP, C4, V2 and βC1 gene fragments were released from the respective recombinant pPCV702SM plasmids and ligated into the *Eco*RI/*Sal*I (*Eco*RI/*Hin*dIII for βC1) sites of pET32a (Merck KGaA, Darmstadt, Germany) to yield constructs pET32-TrAP, pET32-C4, pET32-V2, and pET32-βC1, respectively. This cloning strategy enabled the fusion of the corresponding proteins with an N-terminal thioredoxin (Trx) and histidine (His) tag. The constructs were introduced into *E. coli* [strain BL21-(DE3)] by heat-shock transformation. The transformed bacterial cells were induced using 1 mM IPTG and were grown for 4–6 hours at 28 °C. Cells were lysed with BugBuster^®^ (Merck KGaA, Darmstadt, Germany) supplemented with Bensonase^®^ (Merck KGaA, Darmstadt, Germany; 25 U/mL of lysis buffer) and EDTA-free proteinase inhibitor cocktail (Roche Diagnostics GmbH, Mannheim, Germany). Recombinant proteins were purified by chromatography with Ni^2+^-NTA agarose resin (QIAGEN GmbH, Hilden, Germany), loaded onto 5 mL polypropylene columns (QIAGEN GmbH, Hilden, Germany), and washed with lysis buffer (300 mM NaCl, 50 mM NaH_2_PO_4_ and 20 mM imidazole). Protein fractions were eluted from the Ni-NTA column in elution buffer (300 mM NaCl, 50 mM NaH_2_PO_4_ and 250 mM imidazole). Buffer exchange (20 mM Tris-Cl pH 7.4, 100 mM KCl and 10% Glycerol) was performed using Slide-a-lyzer Dialysis Cassette G2 10K MWCO (Thermo Fisher Scientific GmbH, Bonn, Germany) following the manufacturer’s instructions. Protein samples were concentrated using Amicon Ultra 2 mL centrifugal filters (Merck KGaA, Darmstadt, Germany). Aliquots of recombinant proteins were either frozen as such or dissolved in storage buffer (10mM Tris-Cl pH 7.5, 150 mM NaCl, 0.5 mM DTT, 1 mM EDTA, 50% Glycerol) and stored at −20 °C. SDS-PAGE and western blot analysis of purified proteins are shown in Supplemental Figure S1.

### 2.5. Electrophoretic Mobility Shift Assay (EMSA)

Short ssDNA, ssRNA oligonucleotides and 21/24 nt annealed siRNA were obtained from Sigma-Aldrich (Sigma-Aldrich Chemie GmbH, Seelze, Germany) (Supplemental Table S1). A 139 nt fragment of GFP (acc. no. U87973, coordinates 231–369) was digested with *Pml*I to produce 95 and 45 nt dsDNA. A 175 nt long fragment of the *Cestrum yellow leaf curling virus* CMPS promoter (AF364175) was obtained by PCR with the primers given in Supplemental Table S1. Short ssDNA, siRNA, or ssRNA (400 ng) or GFP or CMPS dsDNA (2–3 µg) were phosphorylated with T4 polynucleotide kinase (New England Biolabs GmbH, Frankfurt, Germany) in the presence of [γ-^32^P] ATP (6000 μCi/mmoL) (PerkinElmer Technologies GmbH & Co. KG, Walluf, Germany). Non-incorporated nucleotides were removed using Quick Spin Sephadex G25 columns (Roche Diagnostics GmbH, Mannheim, Germany). An aliquot of labelled RNA (20–40 ng) or dsDNA 100–200 ng of was incubated with ~800 ng of purified protein in EMSA binding buffer (83 mM Tris-HCl (pH 7.5), 2.5 mM MgCl_2_, 66 mM KCl, 100 mM NaCl, 0.1 M DTT and 0.2% (*w*/*v*) Tween20) at 37 °C for 30 min. The protein-nucleic acid complexes were analyzed in 6% non-denaturing polyacrylamide gels (Anamed Elektrophorese GmbH, Groß-Bieberau / Rodau, Germany) in 0.5× Tris-borate-EDTA [36].

### 2.6. SDS-PAGE

Protein (0.5–2 μg) was added to 10 μL of sample buffer (3× Tris-HCl-SDS) (Thermo Fisher Scientific GmbH, Bonn, Germany) and electrophoresed in 9% SDS polyacrylamide gels in Laemmli buffer. PageRuler Plus Prestained Protein Ladder (Thermo Fisher Scientific GmbH, Bonn, Germany) was used for protein size estimation. Staining with Coomassie Brilliant Blue G-250 (Sigma-Aldrich Chemie GmbH, Seelze, Germany) was used to visualize proteins in gels.

### 2.7. Western Blotting

Western blotting was carried out as described previously [37]. Immunodetection was conducted using a monoclonal anti-His primary antibody (Thermo Fisher Scientific GmbH, Bonn, Germany) and HRP-conjugated rat anti-mouse IgG (Thermo Fisher Scientific GmbH, Bonn, Germany).

## 3. Results

### 3.1. Suppression of S-PTGS

**Figure 1:**
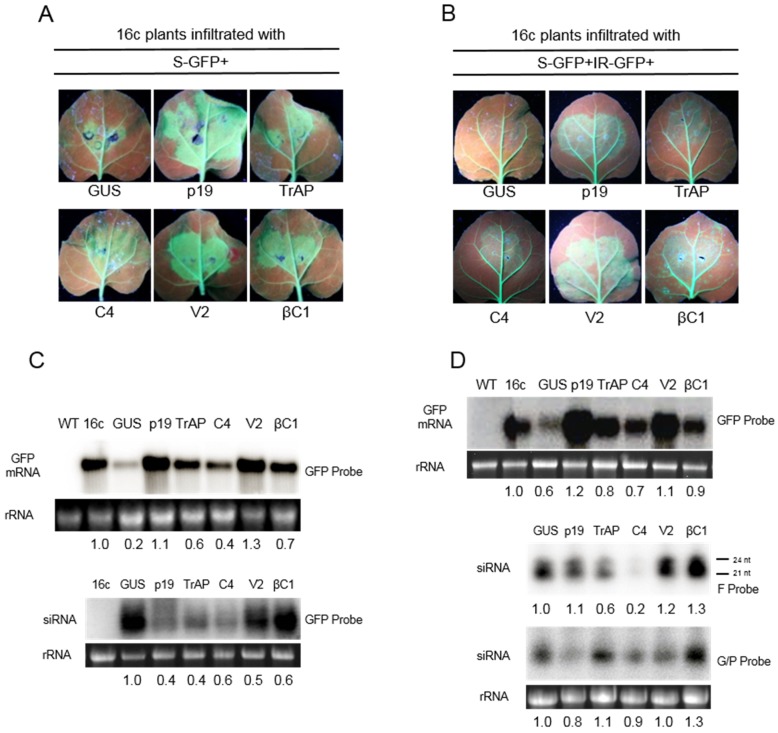
Suppression of gene silencing by proteins encoded by *Cotton leaf curl Kokhran virus* (CLCuKoV) and Cotton leaf curl Multan betasatellite (CLCuMuB). Leaves of *N. benthamiana* 16c plants (16c) in which silencing was induced by infiltration with a construct for the expression of the (sense) green fluorescence protein (S-GFP) gene (**A**) or co-agroinfiltrated with S-GFP and a construct for the expression of an inverted-repeat of the GFP gene (IR-GFP) (**B**); In each case the plants were co-infiltrated with constructs for the expression of either β-glucuronidase (GUS), the p19 protein of TBSV (p19), the βC1 protein of CLCuMuB (βC1), or the transcriptional activator protein (TrAP), C4 protein (C4), or V2 protein (V2) encoded by CLCuKoV, respectively. Plants were photographed under UV illumination at 4 dpi; (**C**) Northern blot of total RNAs extracted from the infiltrated leaf patches of plants described for panels A hybridized with full-length GFP probe for the presence of GFP mRNA (upper panel) or siRNAs (lower panel); (**D**) Northern blot of total RNAs extracted from the infiltrated leaf patches described for panel B probed for the presence of GFP mRNA (GFP probe, upper panel), primary siRNAs (F probe; middle panel) or secondary siRNAs (G/P probe; lower panel). For the Northern blot analyses total RNA extracted from non-infiltrated *N. benthamiana* wild type plants (WT) and a non-infiltrated 16c plants (16c) are shown as controls. Samples were extracted at 4 dpi, the ethidium bromide stained ribosomal RNA (rRNA) band on the gel is shown below the blot to confirm equal loading. The numbers below the blots indicate the relative amounts of GFP mRNA and siRNA after normalization using 16S rRNA as a loading control. The values are given relative to untreated 16c, for GFP mRNA, or 16c co-inoculated with GUS, for GFP-derived siRNA. Image J software was used to estimate image intensities from the images of the blots.

*N. benthamiana* 16c plants (16c) that express the green fluorescence protein (GFP) gene under the control of the *Cauliflower mosaic virus* (CaMV) 35S promoter [38] were co-agroinfiltrated with an *Agrobacterium* culture harbouring a construct for the expression of the full-length GFP gene in sense orientation (pPCV702SM-GFP5) and *Agrobacterium* cultures for the expression of various proteins to be assessed for suppression of sense-PTGS (S-PTGS). Co-agroinfiltration of 16c plants with a construct for the expression of GUS (pPCV702SM-GUS) led to the infiltrated patch showing only weak GFP fluorescence by 4 days post-infiltration (dpi) under UV illumination (Figure 1A). The loss of GFP fluorescence indicates that GUS has no RSS activity and that in the infiltrated patch, decline of GFP expression was due to PTGS. In contrast, the infiltrated patches of 16c plants co-infiltration with a construct for expression of p19 showed intense GFP fluorescence (Figure 1A) indicating that GFP mRNA was not degraded. This is consistent with previous findings that p19 is a strong suppressor of PTGS [39]. Intense GFP fluorescence was also evident for plants co-infiltrated with the construct expressing the V2. For plants co-infiltrated with constructs for expression of βC1, TrAP and C4, the GFP fluorescence was less intense than that for V2 but nevertheless greater than that found for the GUS construct, indicative of RSS activity (Figure 1A).

Northern blot analyses of total RNA extracted from the agroinfiltrated regions of 16c plants at 4 dpi were conducted to detect GFP mRNA and GFP-derived siRNAs (Figure 1C). For plants co-infiltrated with the construct for the induction of silencing and the construct for expression of GUS, the level of GFP mRNA was reduced to 80% that detected in non-infiltrated 16c plants. This was associated with the accumulation of large amounts of GFP-derived siRNAs indicating PTGS. In contrast, co-expression of p19 GFP mRNA increased by 10% and GFP-derived siRNAs decreased by 40%. Similarly, co-infiltration with the V2 construct resulted in a 30% increase in mRNA levels. However, in this case, GFP-derived siRNA levels were 25% higher than for co-infiltration with p19. For co-infiltration with constructs expressing the TrAP or C4, GFP mRNA levels were 25% to 50% lower than for co-infiltration with p19, but 200% to 300% higher than for infiltration with GUS, and were associated with low levels of GFP-derived siRNA. Co-infiltration with the βC1 construct resulted in 30% decrease in GFP mRNA and GFP-derived siRNA levels were 60% to those detected in plants co-infiltrated with the GUS construct. Collectively, these data suggest that TrAP, C4, V2, and βC1 are able to suppress S-PTGS, with V2 being the most potent.

### 3.2. Suppression of IR-PTGS

The ability of the four proteins to suppress IR-PTGS was investigated by co-infiltrating 16c plants with a construct expressing a 139 bp inverted repeat of the GFP cDNA (pPCV702SM-GpG), a construct expressing the full-length GFP cDNA (pPCV702SM-GFP5) and individual constructs for the expression of the virus/satellite proteins (Figure 1B). Analysis of the agroinfiltrated plants under UV illumination at 4 dpi showed no GFP fluorescence for co-infiltration with the GUS construct. In contrast, for all other proteins GFP fluorescence in the infiltrated patches was evident with the strongest fluorescence for V2 and p19 (Figure 1B).

In order to detect GFP mRNA and GFP-derived siRNAs in the infiltrated patches, northern blot analysis was performed (Figure 1D). The F-probe corresponding to one of the repeats of the GpG construct was used to detect primary siRNAs and the G/P probe was used to detect secondary GFP-derived siRNAs (Figure 1D). The data show that primary siRNAs (F probe) were 20% to 30% more abundant in the presence of V2 and βC1 in comparison to samples derived from plants co-infiltrated with GUS construct (Figure 1D, F probe). However, secondary siRNAs were 10% more abundant in the presence of TrAP and 30% more in the presence of βC1 than in all other samples (Figure 1D, G/P probe). For the V2 protein, the levels of secondary siRNAs was reduced by 20% relative to the primary siRNAs. Two classes of primary siRNAs, with sizes of 21 and 24 nt, were clearly detectable. In contrast, only 21 nt long secondary siRNAs were found (Figure 1D, F probe) which is consistent with previous findings [40]. These results indicate that TrAP, C4, V2, and βC1 are able to suppress IR-PTGS (with V2 being the most effective). The results also show that the RSS proteins do not act in the same way, since they significantly differ in the way they affect primary and secondary siRNA production.

### 3.3. Effects of Virus Proteins on Systemic Silencing

**Figure 2:**
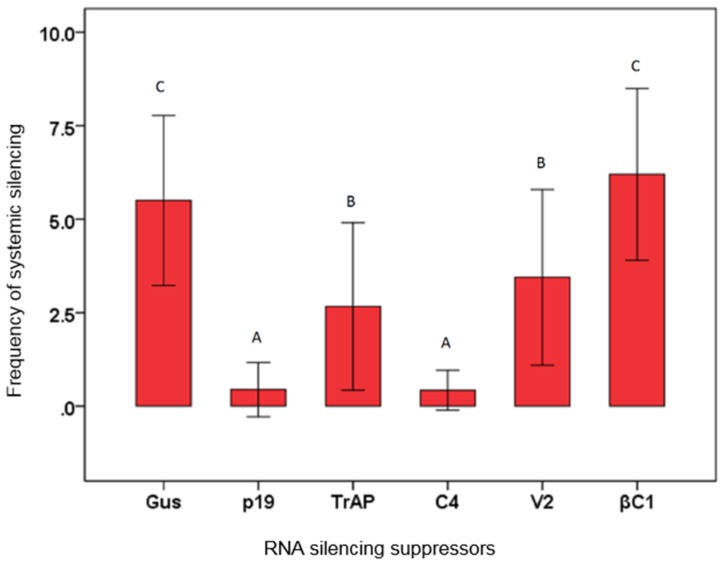
Suppression of systemic silencing. The bar graph shows the percentages of *N. benthamiana* 16c plants exhibiting systemic silencing at 14 dpi with silencing inducer and constructs for the expression of β-glucuronidase (GUS), the p19 protein of TBSV (p19), the βC1 protein encoded by Cotton leaf curl Multan betasatellite, or the transcriptional activator protein (TrAP), C4 protein (C4), or V2 protein (V2) encoded by *Cotton leaf curl Kokhran virus*. The raw data is shown in tabulated form below the graph. The results are of five independent experiments. Plants were examined for systemic silencing at 14 dpi. Error bars represent significant differences in suppression efficiency between the individual constructs and the empty vector in Chi-square tests (*p* < 0.05). A,B,C: Indicates statistically significant differences at the 95% level

RNA silencing spreads cell-to-cell, through plasmodesmata, and may spread systemically through the vascular system of the plant [38,41]. The effects of the four virus/satellite proteins on systemic silencing was assessed (Figure 2). At 14 dpi, 57% of plants agroinfiltrated with either GFP or IR-GFP and the GUS control vector showed silencing in the upper, newly developing leaves which were emerging at the time of infiltration. In contrast, for co-infiltration with the p19 vector only 7% of the plants showed silencing in the upper leaves 14 dpi. This indicates that, as has been shown previously [42], p19 interferes with systemic silencing. The CLCuKoV protein TrAP marginally delayed the establishment of systemic silencing since 44% of the plants showed GFP silencing in apical leaves, whereas 58% systemic silencing was observed in case of V2. However, co-infiltration with the C4 construct resulted in only 5% of plants developing systemic silencing. Unexpectedly, the CLCuMuB βC1 protein appeared to increase the numbers of plants (70%) showing systemic silencing at 14 dpi.

### 3.4. Interactions of Proteins with Nucleic Acids

**Figure 3:**
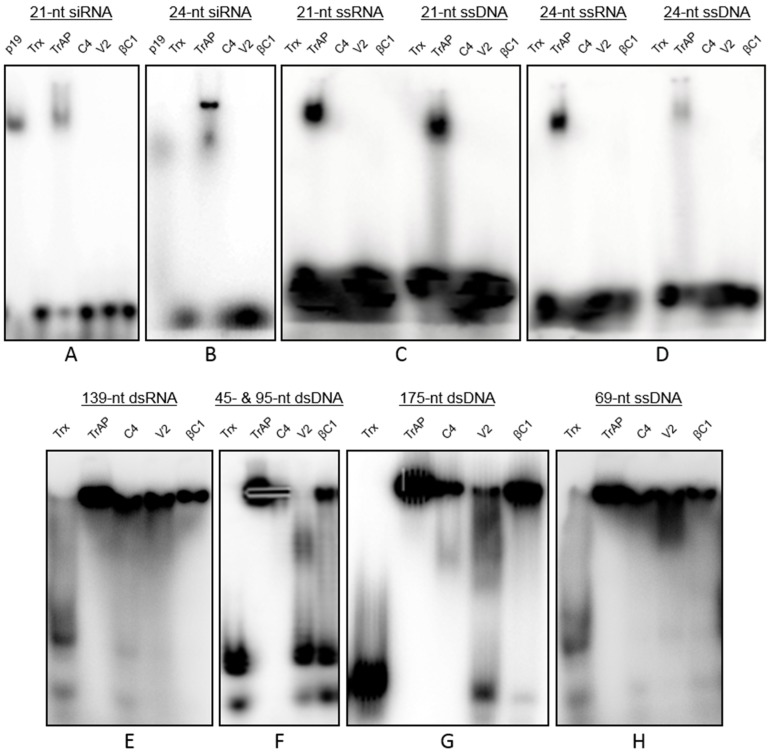
Electrophoretic mobility-shift assays of recombinant Trx-His tagged proteins. In each case, the nucleic acids used in the mobility-shift assays are indicated over the respective blots.

The interactions of viral proteins with nucleic acids was investigated by electrophoretic mobility shift assay (EMSA) using proteins produced in *E. coli*, fused to Trx and His tags, and purified on Ni-NTA columns. The p19 siRNA binding protein (New England Biolabs GmbH, Frankfurt, Germany) was used as a control. The expression and purification of Trx- and His-tagged fusion proteins (TrAP, C4, V2 and βC1) produced a soluble form of the proteins (Supplemental Figure S1A) which were detectable by immunoblot assays (Supplemental Figure S1B). The p19 retarded the mobility of 21 and 24 siRNA-duplexes (Figure 3A,B) as has been reported previously [43]. Similarly, the purified Trx:His-TrAP bound 21 and 24 siRNA-duplexes. However, no binding of 21 and 24 siRNA-duplexes was evident for Trx:His-C4, Trx:His-V2 or Trx:His-βC1 (Figure 3A,B). In assays with 21 and 24 nt ssRNA and ssDNA, only Trx:His-TrAP showed binding (Figure 3C,D). These results indicate that CLCuKoV-encoded TrAP has the capacity to bind to a variety of small nucleic acid molecules.

Assays for the binding of proteins to larger nucleic acid molecules showed that Trx:His-TrAP and Trx:His-C4 bind 45, 95, and 175 nt dsDNA fragments, as well as a 69 nt ssDNA (Figure 3E–H). Trx:His-βC1 strongly bound the 69 nt ssDNA and 175 nt dsDNA but weakly bound the 95 nt dsDNA. Trx:His-V2 appeared to weakly bind the 45 and 175 nt dsDNA but to relatively strongly bind the 69 nt ssDNA. Together, these results show that TrAP is the only CLCuKoV/CLCuMuB protein that binds short RNA and DNA molecules that are either ss or ds. The protein also showed the strongest binding to longer ss and ds nucleic acids. Trx:His-C4 bound all longer nucleic acids. The weakest binding was shown by Trx:His-V2 with a preference for longer nucleic acids whereas Trx:His-βC1 showed a preference for longer nucleic acids (>95 nt) but with no apparent preference for form.

### 3.5. Effects of Transgenic Expression of Protein in Plants

Transgenic plants expressing the CLCuKoV TrAP or V2 genes under the control of the constitutive CaMV 35S promoter developed normally (Supplemental Figure S2A) In contrast, plants harbouring the construct for expression of C4 showed developmental abnormalities reminiscent of virus symptoms (Supplemental Figure S2A). The abnormalities comprised leaf crumpling, upward rolling of the leaf edges, as well as shortening of side branches and petioles.

No transgenic lines were recovered for the CLCuMuB βC1 construct under the control of the 35S promoter. This suggests that βC1 is toxic for the plant. To overcome this problem, *N. benthamiana* was also transformed with the βC1 gene under the transcriptional control of a dexamethasone inducible promoter [32]. *N. benthamiana* lines transformed with this construct showed normal plant development with no evidence of abnormalities. However, when dexamethasone was sprayed on two-month old transgenic plants, the plants developed mild upward curling of leaf edges and mild leaf crumpling (Supplemental Figure S2B). When dexamethasone was sprayed on transgenic plants at the 6–8 leaf stage for three consecutive days, the plants exhibited severe leaf and stem curling, stunting, and mosaic on leaves (Supplemental Figure S2C). The resulting symptoms were persistent; plants remained stunted and did not flower.

**Table:** 

Inoculum	No. of plant systemically silenced/no. of plants inoculated	Silencing(%)
GFP+GUS	27/49	55 ^C^
GFP+p19	2/27	7 ^A^
GFP+TrAP	11/27	41 ^B^
GFP+C4	1/29	3 ^A^
GFP+V2	15/27	56 ^B^
GFP+ βC1	30/44	68 ^C^
IR-GFP+GUS	28/48	58 ^C^
IR-GFP+p19	2/28	7 ^A^
IR-GFP+TrAP	13/27	48 ^B^
IR-GFP++C4	2/30	6 ^A^
IR-GFP+V2	16/26	62 ^B^
IR-GFP+βC1	32/44	73 ^C^

## 4. Discussion

The results presented here show that the investigated CLCuKoV/CLCuMuB proteins exhibit RSS activity. Only the CLCuKoV TrAP bound short nucleic acids, both RNA and DNA, and reduced the accumulation of GFP siRNAs in plants. Vanitharani *et al.* [44] showed the TrAP proteins of *East African cassava mosaic Cameroon virus* (EACMCMV) and *Indian cassava mosaic virus*, but not the closely related *African cassava mosaic virus* (ACMV) and *Sri Lanka cassava mosaic virus*, to similarly reduce siRNA levels *in planta*. Subsequently, the TrAP of EACMCMV was shown not to bind siRNAs [45]. The results here, thus, mark the first identification of an siRNA binding TrAP protein for begomoviruses. This suggests that the CLCuKoV TrAP acts to sequester siRNAs from incorporation into RISC and thereby protect target mRNAs from degradation. Small RNA (sRNA) binding proteins may also bind miRNAs that are important for plant development, thereby likely inducing, at least, some of the symptoms caused by viruses [45]. Consistent with this idea the CLCuKoV TrAP and the closely related CLCuMuV TrAP, and the TrAPs of a number of other viruses, have been shown to induce virus-like symptoms in *N. benthamiana* when expressed from a *Potato virus X*-based vector [46,47].

A number of previous studies have shown begomovirus TrAPs to bind both ss and dsDNA in a sequence non-specific manner [48,49,50]. The results for CLCuKoV TrAP are consistent with this and also, for the first time, show TrAP to bind dsRNA. However, the biological significance of these interactions are unclear. TrAP is a transcription factor required for expression of late viral genes and transactivation of host genes, including genes that negatively regulate RNA silencing [51,52].

The V2 protein of *Tomato yellow leaf curl China virus* (TYLCCNV) binds 21 and 24 dsRNA duplexes and 24 nt ss RNAs but no 21 nt ssRNAs [53]. This contrasts with the results here showing CLCuKoV V2 not to bind sRNAs, but is in agreement with the results obtained with CLCuMuV [26]. As well as being an RSS, the V2 of monopartite begomoviruses has been implicated in movement [54] which might explain the interaction of the protein with long, preferentially ssDNA. However, it has recently been suggested that V2 RSS activity overcomes a host RNA silencing-based resistance to virus movement rather than having actual movement function [55].

The results obtained here show CLCuKoV C4 not to bind sRNAs or small DNAs (sDNAs) but to bind long dsRNA and both long ds and ssDNA. In previous studies, the CLCuMuV C4 was shown to bind short RNAs with a preference for ds forms [26]. Although the AC4 protein of EACMCMV appeared to lack any sRNA binding capacity, the ACMV AC4 bound sRNA [45].

Here, the βC1 protein of CLCuMuB was shown to bind long ss and dsDNA and long dsRNA but no sRNAs or sDNA. This is in general agreement with previous studies of the same βC1 protein showing poor binding of short ssRNA [26], and of the Tomato yellow leaf curl betasatellite βC1 binding both, long ss and long dsDNA [25]. The findings here are also in agreement with the results of Tiwari *et al.* [56] who showed sequence non-specific binding of the βC1 protein of another CLCuMuB isolate but concluded that their data showed “sequence-specific” DNA binding. The biological significance of DNA binding for βC1 remains unclear but it could be related to the proposed function of βC1 in viral cell-to-cell movement [57].

Using the βC1 from the same CLCuMuB clone as was used in the study here, Eini *et al.* [58] concluded that the protein was able to prevent systemic silencing; contrasting with our results. Eini *et al.* [58] used two techniques, including grafting, to investigate the inhibition of systemic silencing. It is possible that the more simple technique used here did not detect the effects of the CLCuMuB βC1 on systemic silencing. However, the finding that CLCuMuB βC1 appeared to enhance systemic silencing is consistent with what is known about the βC1 of other betasatellites. The βC1 of Tomato yellow leaf curl China betasatellite (TYLCCNB) has been shown to interact with and to inhibit S-adenosyl homocysteine hydrolase (SAHH). Downregulation of SAHH prevents local silencing, but enhances systemic silencing [59]. Thus a βC1 induced reduction in SAHH activity could potentially lead to stronger systemic silencing.

Of the viral proteins analyzed here, only the C4 protein efficiently inhibited systemic silencing. The potency for C4 preventing systemic silencing appeared to be stronger than that of p19. The nature of the systemic silencing signals remains unclear. In *Arabidopsis*, the production of systemic silencing signals were independent of DCL1, DCL2, DCL3, DCL4, RDR2, and RDR6, indicating that the signal molecule may not be a typical sRNA [15]. However, reception of systemic silencing signals requires RDR6, RDR2, POLIV, and DCL3, suggesting that a nuclear RNA silencing step is important at this stage [15]. Since the mechanistic details of the production and reception of systemic silencing signals are still elusive, it is difficult to draw any firm conclusion regarding the mode of action of C4 in interfering with systemic silencing. Previously the TrAPs of *Tomato golden mosaic virus* and *Mungbean yellow mosaic virus* [51,60], the V2 protein of TYLCCNV [53] and the βC1 protein of CLCuMuB [58] have been shown to inhibit systemic silencing. This is the first identification of a begomovirus C4 protein having strong systemic silencing suppressor activity.

The proteins differed in their potential to suppress S- and IR-PTGS, respectively. Although TrAP was the only protein examined here that bound sRNAs, it only marginally reduced primary siRNAs and had no effect on the accumulation of secondary siRNAs. In contrast, the V2 protein had no effect on the accumulation of primary siRNAs but significantly reduced the levels of secondary siRNAs. The V2 protein of *Tomato yellow leaf curl virus*-Israel interacts with SUPPRESSOR OF GENE SILENCING 3 (SGS3) [61] which, together with RDR6, is required for transitivity [62]. Thus V2-mediated reduction of secondary siRNAs may be due to the decrease in RDR6-mediated dsRNA production by interacting with and impairing the function of SGS3.

RSS proteins may interfere with normal plant development, particularly if they affect the miRNA pathway [63]. Although all the proteins analyzed here inhibited silencing, only the C4 and βC1 proteins induced developmental abnormalities that resemble the symptoms produced during viral infection. Surprisingly, TrAP, the only protein binding sRNAs, which thus may possibly also bind miRNAs, induced no phenotypic changes upon overexpression in *N. benthamiana*.

## 5. Conclusions

Overall the results presented here show that CLCuKoV, in common with CLCuMuV, encodes a number of RSS proteins. Although four proteins with RSS activity were identified here, the complex could include as many as six RSS proteins with the demonstration that the viral and alphasatellite-encoded Rep proteins may have RSS activity [64,65,66]. It is clear that the four RSS proteins examined here differ in their activities with TrAP likely sequestering siRNAs and the C4 protein inhibiting the spread of systemic silencing signals. It is also evident that homologous proteins from even quite closely related begomoviruses may differ in their RSS activity and their mechanism of action. This, at least in part, explains why begomoviruses are such successful and destructive pathogens, with the ability to complement functions by synergism, recombination, and pseudo-recombination (component exchange) to adapt to new environmental niches.

## Supplementary Files

Supplementary File 1
