# Supplementary material for: Functional Analysis of Cotton Leaf Curl Kokhran Virus/Cotton Leaf Curl Multan Betasatellite RNA Silencing Suppressors
Source: Biology (Basel). 2015 Oct 23;4(4):697–714. doi: 10.3390/biology4040697 (PMC4690014; doi:10.3390/biology4040697)
Supplement: Supplementary File 1 [file biology-04-00697-s001.pdf]

Supplemental Materials

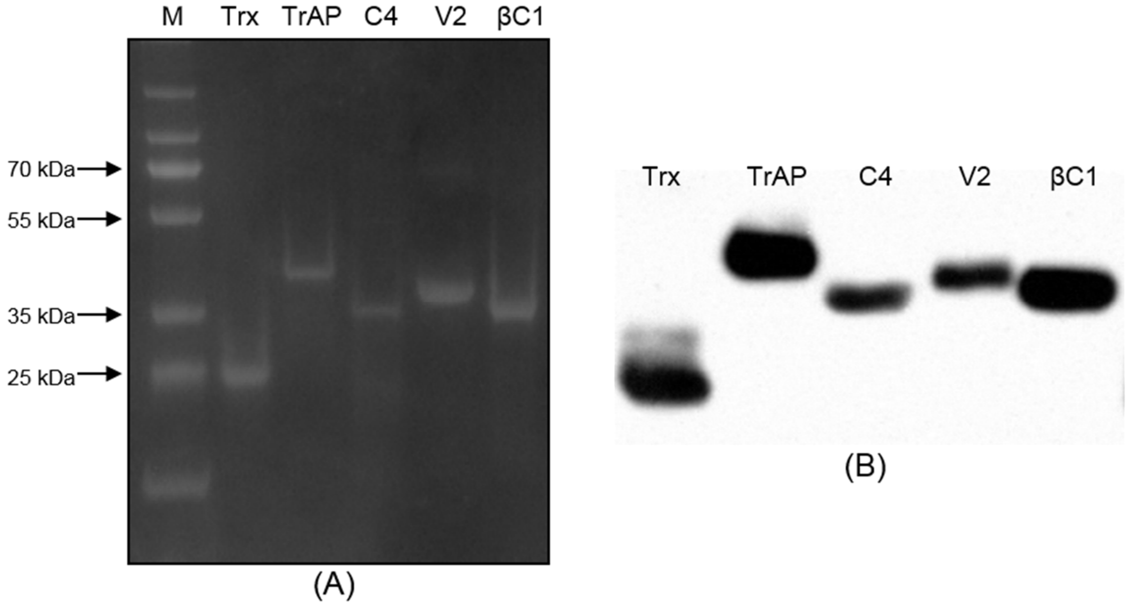

Figure S1. Analysis of purified, *E. coli* expressed, thioredoxin- (Trx) and histidine- (His) tagged fusion proteins by denaturing SDS polyacrylamide gel electrophoresis (SDS-PAGE). (**A**) Coomassie blue stained SDS-PAGE gel containing samples (3 µg) of soluble protein fractions after Ni-NTA purification. (**B**) Western blot of a duplicate gel of that in panel A probed with an anti-His tag antibody. The samples run on the gels were thoredoxin (Trx), the βC1 protein encoded by Cotton leaf curl Multan betasatellite, βC1, the transcriptional activator protein (TrAP), C4 protein (C4) and V2 protein (V2) encoded by *Cotton leaf curl Kokhran virus*.

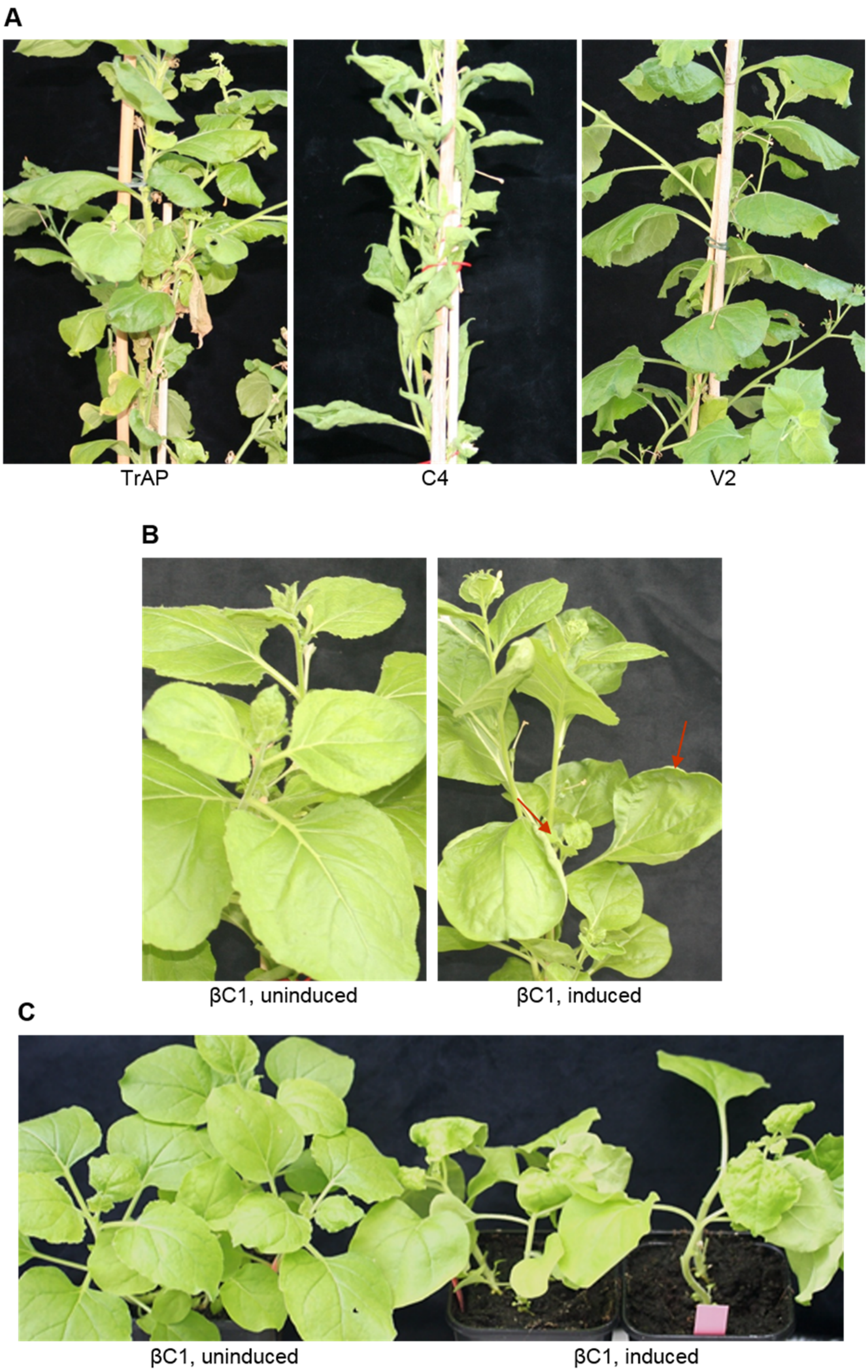

Figure S2. (**A**) Transgenic *N. benthamiana* plants harbouring constructs for the expression of *Cotton leaf curl Kokhran virus* TrAP, C4 and V2 under the control of the *Cauliflower mosaic virus* 35S promoter. (**B**) *N. benthamiana* plants transformed with a construct for the expression of Cotton leaf curl Multan betasatellite βC1 under the control of a dexamethasone inducible promoter. The phenotypes of plants following dexamethasone induction of βC1 expression are shown for induction of (**B**) two month old plants and (**C**) plants at the 6-8 leaf stage (sprayed with dexamethasone for 3 consecutive days). Arrows indicate leaf curling in panel B.

Table S1. Oligonucleotides used in the study.

**Oligo**	**Sequence (5' to 3') ***
C2-EcoRI-F	GGCGAATTCATGCAATCTTCATCACCCT
C2-SalI-R	ATATGTCGACCTAAAGACCCTTAAGAAACG
C4-EcoRI-F	GGCGAATTCATGGGACTCCTCACTTGC
C4-SalI-R	GATGTCGACCTAGTTCCTTAATGACTCTA
V2-EcoRI-F	GGCGAATTCATGTGGGATCCACTGTTAA
V2-SalI-R	TATGTCGACCTAGGAACATCTGGACTT
βC1-EcoRI-F	GGCGAATTCATGACACCGAGCGGAACA
βC1-XhoI-F	GGCCTCGAGATGACACCGAGCGGAACA
βC1-HindIII-R	GGCAAGCTTTTAAACGGTGAACTTTTTATTG
βC1-SpeI-R	GGCACTAGTTTAAACGGTGAACTTTTTATTG
CMPS-F	ATCCTGGCAGACAAAGTGG
CMPS-R	GAAGTAGGATCTCTAGAA
GFP-G-F	AGTAAAGGAGAAGAACTTTTCAC
GFP-G-R	TGATCTGGGTATCTTGAAAAGC
GFP-F-F	TATGAAGCGGCACGACTTC
GFP-F-R	GATCCTGTTGACGAGGGTG
GFP-P-F	GAGCTTAAGGGAATCGATTTCA
GFP-P-R	TCGTTGGGATCTTTCGAAAGG
GFP-FL-F	ATGAAGACTAATCTTTTTCT
GFP-FL-R	TAAAGCTCATCATGTTTGTA
21nt siRNA	AGAGUGCCAUGCCCGAAGGUU
21nt DNA	AGAGTGCCATGCCCGAAGGTT
24nt siRNA	AGAGUGCCAUGCCCGAAGGUUAUU
*Sal*I-*Xba*I-HA-Tag	GCGTCGACGTCTTGCTCTAGAGATGTATCCATATGATGTTCCGGATTACGC GGAACGAGCTATACAAGG

* In each case the introduced restriction endonuclease recognition site is underlined.
